# Approaches to Recording Drug Allergies in Electronic Health Records: Qualitative Study

**DOI:** 10.1371/journal.pone.0093047

**Published:** 2014-04-16

**Authors:** Bernard Fernando, Zoe Morrison, Dipak Kalra, Kathrin Cresswell, Aziz Sheikh

**Affiliations:** 1 eHealth Research Group, Centre for Population Health Sciences, The University of Edinburgh, Edinburgh, United Kingdom; 2 Centre for Health Informatics and Multiprofessional Education, University College London, London, United Kingdom; 3 The School of Health in Social Science, The University of Edinburgh, Edinburgh, United Kingdom; 4 Allergy and Respiratory Research Group, Centre for Population Health Sciences, The University of Edinburgh, Edinburgh, United Kingdom; 5 Harvard Medical School & Harkness Fellow in Health Care Policy and Practice, Division of General Internal Medicine and Primary Care, Brigham and Women's Hospital/Harvard Medical School, Boston, Massachusetts, United States of America; Universidad de Valladolid, Spain

## Abstract

**Background:**

Drug allergy represent an important subset of adverse drug reactions that is worthy of attention because many of these reactions are potentially preventable with use of computerised decision support systems. This is however dependent on the accurate and comprehensive recording of these reactions in the electronic health record. The objectives of this study were to understand approaches to the recording of drug allergies in electronic health record systems.

**Materials and Methods:**

We undertook a case study comprising of 21 in-depth interviews with a purposefully selected group of primary and secondary care clinicians, academics, and members of the informatics and drug regulatory communities, observations in four General Practices and an expert group discussion with 15 participants from the Allergy and Respiratory Expert Resource Group of the Royal College of General Practitioners.

**Results:**

There was widespread acceptance among healthcare professionals of the need for accurate recording of drug allergies and adverse drug reactions. Most drug reactions were however likely to go unreported to and/or unrecognised by healthcare professionals and, even when recognised and reported, not all reactions were accurately recorded. The process of recording these reactions was not standardised.

**Conclusions:**

There is considerable variation in the way drug allergies are recorded in electronic health records. This limits the potential of computerised decision support systems to help alert clinicians to the risk of further reactions. Inaccurate recording of information may in some instances introduce new problems as patients are denied treatments that they are erroneously believed to be allergic to.

## Introduction

Adverse drug reactions are very common [1]; they now, for example, account for an estimated 6.5% of hospital admissions in the United Kingdom (UK) [2]. The risk of one or more adverse drug reactions (i.e. responses to medical products that are noxious and unintended) occurring as a result of drugs initiated at the time of admission to the hospital or continued in hospital has been estimated at 14.7%, of which just over half are judged to be possibly or definitely avoidable [3]. An estimated 0.7–2.3% of deaths following adult emergency admissions with adverse events (i.e. undesirable events experienced by patients whilst taking medicines) are attributed to treatment in primary care [4].

Drug allergies represent an important subset of adverse drug reactions (see Table 1). These are of particular interest because these can result in life-threatening reactions and are often preventable, particularly in the context of managing those with known drug allergies. Clinical computerised decision support systems (CDSS) in prescribing modules are widely seen as having considerable potential to reduce the risk of allergic reactions to drugs by drawing on information held in electronic health records (EHRs) to generate tailored alerts in real-time [5]–[7]. This is because CDSS have the potential to reduce prescribing errors and thus repeat exposure to drugs (or similar classes of drugs) for which a drug allergy is already recorded in the system. CDSS tools are now widely used in primary care in the UK, and are increasingly being made available in hospitals both in the UK and internationally [8], [9]. Irrespective of the setting, these CDSS are crucially dependant on the availability of accurate clinical information in coded format so as to enable the underlying algorithms to successfully operate [10].

**Table 1:** Definitions.

**Adverse drug reaction**
An adverse drug is a response to a medicinal product, which is noxious and unintended. It is also known as a side effect. http://www.adrreports.eu/EN/glossary.html
**Allergic drug reaction**
Allergic drug reaction is an adverse drug reaction. It is an immunologically mediated reaction characterised by specificity, transferability by antibodies or lymphocytes, and recurrence on re-exposure. Vervloet D, Durham S (1998) Adverse reactions to drugs. BMJ 316: 1511–1514

Understanding how drug allergies are currently recorded is therefore important as such information can inform deliberations on how to realise the potential offered by the prescribing decision support systems that are now increasingly embedded in EHRs. This study aimed to address an important gap in the documented knowledge of current practices of recording adverse drug reactions and in particular drug allergies in EHRs. We were commissioned by England's Department of Health to undertake an investigation into current recording practices of drug allergy and other adverse drug reactions in order to inform deliberations on how to enhance patient safety.

## Materials and Methods

### Design

CDSS used in prescribing modules are an important example of what are sometimes known as eHealth interventions [11]. They are now often designed and evaluated as sociotechnical interventions [12] rather than purely technological innovations as was the case in the recent past. Due consideration to human and organisational factor considerations is crucial to the successful implementation and adoption of these systems [13], [14]. We sought to investigate recording of adverse reactions and the interplay between social and technical aspects of this process by exploring behaviours in context using a qualitative case study approach [15]. This approach allows an in-depth multifaceted exploration of complex issues in their real-life settings. The process of recording allergic drug reactions electronically, and the value derived from recording, was conceptualised as a case and investigated using a combination of interviews, documentary analysis and observations.

### Ethics and governance

We received ethical approval for this work from the National Research Ethics Service – Brighton West Ethics Committee (MREC Ref: 10/H1111/25). The research team obtained site-specific permissions from local Research and Development offices, facilitated by the UK Clinical Research Network (UKCRN) and the Primary Care Research Network (PCRN). Written informed consent was sought from all participants using an approved consent form. Where participants chose to be interviewed by telephone without forwarding a completed consent form, verbal consent was recorded and transcribed verbatim and this fact was recorded in accordance with the protocol approved by the Ethics Committee. All data were anonymised to protect the confidentiality both of organisations and individual participants.

### Settings

GP practices in South East of England were selected for site visits. They were small in size with 1 to 5 GPs for a practice. The participants of a group discussion were from the Allergy and Respiratory Expert Resource Group of the Royal College of General Practitioners who were from a variety of GP practice settings. Secondary care clinicians interviewed were from large academic hospitals in the UK.

### Sampling and recruitment

We purposively selected stakeholder roles that represented multiple scenarios of adverse drug reaction recording and use. We initially approached interviewees with an interest or expertise in drug allergy and/or adverse drug reactions through personal networks. We then snowballed [16] from this initial sample on the basis of suggestions made by interviewees. Potential interviewees were sent an email invitation that included information about the research and a consent form. A single email reminder was sent to those who did not respond to the initial invitation after three weeks. Primary and secondary care clinicians, nurses and pharmacists, clinical pharmacologists, informatics and industry specialists, and managers working within the drug regulatory sector took part in the interviews, group discussion and observations. Among interviewees, there were four GPs, three pharmacists, one nurse, three clinical pharmacologists, three secondary care physicians (one anaesthetist, one junior hospital physician and one paediatrician), two managers from the medicines regulatory sector and one industry expert. The four site visits were to GP practices with different clinical systems. They were selected for the site visits, since the most developed UK recording systems were those that found in primary care. We also interviewed the secondary care clinicians as they could offer a different context such as challenges of using less advanced computer systems for recording adverse reactions. The variety of stakeholders allowed us to gain an insight into frontline recording practices in a range of settings, as well as underlying drivers.

### Data generation and handling

Semi-structured interviews were the principal data source and were employed to understand and obtain insights into perspectives on and experiences of clinical documentation of adverse drug reactions. Participants were asked about drug allergies and adverse drug reactions separately while appreciating their relationship mentioned above. Interviews were transcribed and checked by the researcher for any errors, which were corrected. Interviews and observations of relevant recording practices by GPs were made during site visits and relevant screen images were captured. A group discussion was held on approaches to accurate coding of allergy with members of the Royal College of General Practitioners' Allergy and Respiratory Expert Resource Group.

### Data analysis

Data analysis was based on an appreciation of sociotechnical principles [17] in evaluation of eHealth innovation. Data collection and analysis took place concurrently, allowing us to modify the topic guide for the future interviews and feeding back emerging themes into subsequent data collection (e.g. by searching for disconfirming evidence). Transcribed data from interviews, the observations and field-notes from the discussion group were read repeatedly by the researcher (BF, a clinician with an interest in structured documentation), and emerging themes were identified and coded for analysis. This was achieved by examining the underlying tensions and emerging common themes within data sources initially and then by comparing these across data sources to produce a coherent account. Results were discussed with the wider multi-disciplinary research team, allowing alternative explanations for the findings and emerging themes to be explored in detail. The main themes emerging were judged to be converging towards saturation when no new themes emerged across data collection activities.

## Results

The final dataset comprised of 21 (i.e. four face-to-face and 17 telephone) interviews, observations made in four site visits, and notes made during an expert discussion group involving 15 clinicians. Five key themes emerged from analysis of our data, namely the: (1) diverse approaches to recording of drug allergies (and more generally adverse drug reactions); (2) variable extent of recording; (3) perceived benefits of recording; (4) perceived risks of recording such information; and (5) the wider contextual considerations including the training of professionals, incentives and secondary uses of data.

### Diverse approaches to recording of drug allergies

We found diverse approaches to recording drug allergy. All GPs interviewed preferred to record this information in the electronic record themselves rather than delegate this responsibility to other members of the team, because they recognised the importance of accurate documentation.

*“…I feel it is very important issue to record patient's allergy and it has to be 100% accurate therefore I thought that it is my role as a clinician to enter all the allergy, and take sole responsibility for entering, and to date nobody else enters allergy but me.”* (Interview 11, GP/observation)

In contrast, in hospitals, a wide range of professionals (e.g. doctors, nurses and pharmacists) were involved in the recording of the drug allergy and adverse drug reactions.

*“Absolutely the current situation is a combination of different healthcare professionals. Basically nursing staff, medical staff, and pharmacy staff can record allergies and adverse reactions on our system. Probably the nursing record the highest percentage followed by pharmacists and then by medical staff.”* (Interviewee 2, Pharmacist, Secondary care)

In primary care, we observed that templates or pick lists of terms were used to record drug allergy and adverse drug reactions. We also observed that templates (where data entry fields were bound to pre-assigned clinical codes) were commonly used for structured recording. Participants reported that although templates allowed quick data entry and automatic coding, they provided limited opportunity for recording contextual information, which was typically captured within an accompanying free-text narrative to aid interpretation of coded terms.

*“…I think sometimes it forces you to adapt your history to fit the boxes. But at the same time in lots of ways I think it is better to have structure because otherwise everyone will be putting very different things down and some people may record information than others so that at least it gives a sort of minimum level of information.”* (Interviewee 11, GP)

The full dataset of an example GP template with attributes constructed from various templates reviewed is shown in Table 2.

**Table 2:** Data set of an example GP system template used for drug allergy or drug intolerance.

Drug allergy/Drug intolerance		
	Clinician	[Name]
	Drug name	[Name]
	Read term for the reaction/	[Read code]/
	Read term for the allergic reaction	[Read code]
	Reaction type	Adverse effect
		Allergy
		Intolerance
	Date of recording	[Date/Time]
	Severity	Minimal
		Mild
		Moderate
		Severe
		Very severe
		Potentially fatal
	Notes	[Free-text narrative]
	Certainty	Tentative
		Unlikely
		Possible
		Likely
		Certain
		Absolute

GPs were divided in their views on editing or removing incorrect records in electronic systems. Some GPs said they deleted incorrect drug allergy records to prevent false alerts, while others argued against this practice.

*“I do remove because otherwise we get a warning every time which isn't necessary because it's no longer valid.”* (Interviewee 11, GP)

*“I do have a dilemma of removing it because at the time it was a valid problem so I'm not removing an historic problem even though it's not valid now, but it was important at the time of… but there's no other way around that.”* (Interviewee 4, GP)

We observed that severity of reaction could be documented in some templates; this was however perceived to be clinically unhelpful by several participants as grading of severity was often subjective and not necessarily a reliable guide to the severity of future reactions.

Although the primary reason for recording drug allergy and adverse drug reactions was for direct patient care, in some situations this information was also recorded with secondary uses in mind – for example. pharmacovigilance by regulatory agencies such as the UK Medicines and Healthcare Products Regulatory Agency (MHRA). However participants reported that different coding systems (i.e. Read codes, SNOMED-CT, DM&D and MedDRA) were used by different organisations (i.e. primary, secondary care and regulatory agencies). When data from multiple databases were used in pharmacovigilance investigations, participants stated that code mapping tables were needed to aggregate data between multiple systems.

*“…SNOMED is for medical information and DM&D for product information, two terminologies that are not used in medicines regulation. There are approaches to develop a mapping where when we receive the electronic file from the general practice that we identify the SNOMED code and have it translated electronically into the MedDRA code and that we identify the DM&D code and translate that into the appropriate drug within our pharmacovigilance drug dictionary. We need to do this to avoid manual resource being used re-coding thousands of messages.”* (Interviewee 15, Manager, MHRA)

### Variable extent of recording

We found when a drug was discontinued because of drug allergy or adverse drug reaction the reason for discontinuation was not usually recorded in computer systems. The reason for discontinuation of a drug is important information that needs recording because it provides the context of the reaction. Clinicians reported difficulties in distinguishing between allergy, other adverse drug reactions, intolerances and other side effects.

*“A lot of clinical staff struggles to make a difference between allergy [and] adverse drug reactions.”* (Interviewee 7, Pharmacist, Secondary care)

Perhaps as a result of this, the participants felt the recording of drug allergy and other adverse drug reactions was often incomplete.

*“Well I think the biggest challenge is first of all whether it is recorded.”* (Interviewee 8, Academic Pharmacologist)

This was at least in part attributed to the fact that recognition of drug allergy and other adverse drug reactions was considered difficult. None of the participants reported that they used scoring tools [18] to help identify drug allergy or adverse drug reactions. Taking a detailed history of the reported reaction was, however, considered by participants to be very important and this was therefore the recommended approach to clinical diagnosis. In some hospital systems we observed, the presence of drug allergy was not coded and this information could therefore only be displayed back to the user as a free-text entry. As a result, this information was not computable and not usable in CDSS for decision support.

*“And if they have no known drug allergies then that's the one entry that's codified. So basically when they go on to the allergy recording screen, if they've no known drug allergies they can select that automatically. But if they have an allergy or an adverse reaction then they then they've got to type the information that associated with it.”* (Interviewee 12, Pharmacist, Secondary Care)

We observed that in some GP systems, observed drug allergy and adverse drug reactions could be recorded as a class effect (e.g. penicillins or tetracyclines) while in other systems this could only be recorded as individual drug reactions (e.g. phenoxymethylpenicillin or oxytetracycine). Several GPs interviewed were often unsure of the fact that the outputs from their prescribing CDSS would be influenced by how information was recorded. That said, the GPs felt the systems worked satisfactorily for them. Participants reported that, in hospitals, specially trained coders read and interpreted clinical notes for coding important concepts.

### Perceived benefits of recording relevant information

We found a widespread appreciation of the need for the accurate recording of drug allergy and adverse drug reactions. This was particularly motivated by the potential safety gains from computerised prescribing decision support.

*“I think the main issue is about documenting them in a coded way so that if you were to prescribe that drug or a drug in the same class again that you would actually get a warning. I think that is most important things.”* (Interviewee 4, GP)

One participant reported that some innovative decision support tools had been piloted [19] – for instance, when drug allergy and adverse drug reactions were suitably coded, intelligent pick lists for prescribing drugs were possible as shown below.

*“So you know rather than…necessarily showing all the details it's sort of only as if were systems where permissive and straight took you away from drugs where there may have been drug reactions or allergies in the past and put them into the bottom of the [pick] list. So turned the decision support on its head.”* (Interviewee 3, Secondary Care Physician)

### Perceived risks of recording this information

We noted that although different healthcare professionals recorded drug allergy and adverse drug reaction information in electronic systems, not all followed the same diagnostic criteria, which often resulted in inaccurate information.

*“Elsewhere I have worked with electronic prescribing; nursing staff have also recorded information. And that led to particular problems when you think about things like diarrhoea and so on with penicillin. That's not an allergy, clearly.”* (Interviewee 2, Pharmacist, Secondary care)

Perhaps, as a result of this and the practice of multiple recording of this information, the participants reported the recording of adverse drug reactions as often being inconsistent, inadequate and incomplete.

*“I think it's… actually the documentation is actually quite poor, allergies and adverse reactions. And one reason for that is that there's not a single record…most patients' have multiple records and the information isn't consistent across the records.”* (Interviewee 6, Anaesthetist, Secondary care)

We observed that a coded record of drug allergy or adverse drug reaction was therefore not always an accurate record. Participants suggested that a suspected drug allergy or suspected adverse drug reaction was often recorded as a (implicitly definite) drug allergy or adverse drug reaction, which resulted in an inability to distinguish confirmed diagnosis (whenever this was possible) from the majority of suspected reactions in the coded record. Participants felt that “Drug allergy” was thus in some respects a widely used and perhaps misused term as not all recorded drug allergies were clearly established.

*“The trouble is that allergy you know is a very you know widely used and misused word. You know a lot of people who have an adverse drug reaction you know believe that it's an allergy but of course it isn't in true terms, true immunological terms an allergy.”* (Interviewee 8, Academic Pharmacologist)

Participants stated that adverse drug reactions were often incorrectly recorded as drug allergy with unintended consequences for future prescribing; for example, prescribing of alternatives to the antibiotic of choice when a suspected drug allergy to penicillin was recorded.

*“The trouble is if the…there can be a downside to that in that if people are recorded as having adverse reactions to drugs which they haven't in fact had an adverse drug reaction to then you know that can prevent a potentially important treatment being given to patients. You know you think of patients who are wrongly recorded as being allergic to penicillin not then being given penicillin when it's clearly the best treatment on a future occasion.”* (Interviewee 8, Academic Pharmacologist)

### Wider contextual considerations: training, incentives and secondary uses of data

Primary care-based participants reported that they did not have formal training on how to record adverse drug reactions, whilst participants working in some hospitals said that where relevant, they were offered training.

*“As in most things with GPs we just try things don't we, we just teach ourselves quite often, no one taught me.”* (Interviewee 11, GP)

*“Everybody that uses the system has a training programme and they are shown what to do… junior doctors actually get feedback on their performance based on alerts that fire off.”* (Interviewee 2, Pharmacist, Secondary care)

We noted that recording of adverse drug reactions in clinical systems for direct patient care and for secondary use presented different scenarios. In the first instance, future risk of a reaction and, in the latter detailed contextual information of the reaction, were recorded.

*“…you are trying to do very different use cases. In clinical care, you are trying to run decision support…In pharmacovigilance you are trying to collect to find adverse reactions not just allergies, most particularly in medicines surveillance.”* (Interviewee 12, Research Pharmacist)

Both scenarios reflected unscheduled (or spontaneous) recording of information and there was no mandated scheduled (or routine) recording of adverse drug reactions. Participants reported that neither activity was incentivised in the UK.

Some participants felt that incentives for recording this information were not a good idea. Patient safety and professional standards should, the participants felt, to be the drivers for recording or reporting adverse drug reactions.

*“No. I mean I think incentivising would not necessarily be you know would be…I don't think would be a good thing. I do actually think that there should be some professional responsibility.”* (Interviewee 8, Academic Pharmacologist)

## Discussion

### Summary of main findings

There was widespread acceptance among healthcare professionals of the need for accurate recording of drug allergy and the motivation for this was the potential safety gains achieved when prescribing CDSSs are used. This helps to explain why drug allergy alerts are less likely to be over-ridden than other forms of prescribing alerts [22] [23]. However, although recognised as having different aetiologies, drug allergy and adverse drug reactions were treated as synonyms in practice. The primary reason being accurately diagnosing and distinguishing between drug allergy and adverse drug reactions can be difficult outside specialist facilities. These circumstances therefore lead to widespread recording of suspected drug allergy and suspected adverse drug reactions under the generic heading of “drug allergy” [19], an approach that fails to enable optimal leverage of prescribing CDSS [24].

This work has furthermore highlighted the importance of wider contextual considerations, these including the ambiguity of clinical diagnosis, the extent of the lack of diagnostic facilities, the role of incentives and deficiencies of current recording systems. Our findings showed that terms such as ‘drug allergy’ and ‘adverse drug reactions’ are used in practice with disregard to their formal definitions (Table 1). There is a tendency; it seems, for overuse of the term ‘drug allergy’. Given the professional buy-in, the already substantial recording, the opportunity to share structured data throughout the NHS, and the major investments in prescribing CDSS still taking place, this represents a ripe, clinically important area for future research.

### Strengths and limitations of this work

We purposively sampled those with an established interest in this area, but also front-line clinicians and industry representatives; hence we have been able to understand this issue from a broad range of perspectives. These interview-based data were supplemented by observations made during site visits to GP practices, data collected during a group discussion with the Allergy and Respiratory Expert Resource Group of the Royal College of General Practitioners and by reviewing relevant publications [2], [3], [19]–[24], all of which helped us to contextualise and triangulate findings. Interpretation of data was aided by discussions amongst members of our multi-disciplinary group. The final few interviews failed to generate any major new insights this indicating that saturation had been achieved.

Data collection was carried out by the lead author (who is a practising GP with a health informatics background) and whilst this clearly facilitated the relevance of our work for existing clinical practice, this influenced data generation and analysis. The multi-disciplinary data analysis discussions however allowed for wider reflection and input into data analysis. The site visits were confined to one region in England and this may limit the transferability of findings to other settings and clinical contexts. This issue needs to be investigated through follow-on work. Detailed observation of allergy documentation during site visits and analysis of allergy records in GP systems for completeness and consistency were not possible due to time and patient confidentiality related constraints. They were limitations for triangulation of data collected during analysis and should be addressed in future research.

### Conclusions

It is important to accurately record the name of the suspected drug, a description of observed reaction, future risk and any contextual contributory factors when a patient is suspected of having drug an adverse drug reaction in order to leverage the benefits of prescribing CDSS. The information recorded and coded in practice varies depending on the purpose of recording and the nature of the tools available for recording. Although healthcare professionals widely appreciate the importance of recording this information there is at present no agreement amongst clinicians on what needs to be recorded in EHRs and how. The current approach to recording this information is thus inconsistent between primary and secondary care, even though this information increasingly needs to be sharable between care providers in the context of care for individual patients and for aggregation for monitoring purposes. As record sharing becomes routine across the NHS, for example through shared medical summaries, the aggregation of inconsistently captured drug allergy data may introduce the risk of erroneous prescribing recommendations.

A standard terminology used throughout clinical practice for this documentation needs to be complemented by standardised templates and user interface tools that encourage consistent and high quality recording of information on drug allergies. Efforts now need to be focused on improving professional standards in diagnosis, documentation and reporting of drug allergies and adverse drug reactions. This should be informed by further research to develop comprehensive data sets and situation specific terminology subsets. This will be crucially dependant on improved capability for investigation of suspected drug allergy or an adverse drug reaction. Our work strongly suggests that frontline clinicians would welcome such developments, which are, it is believed, likely to reduce risk of prescribing-related iatrogenic harm. This is an area that it is important in particular for future iterations of drug allergy guidelines to address [25].
